# Recombinant production of self-assembling β-structured peptides using SUMO as a fusion partner

**DOI:** 10.1186/1475-2859-11-92

**Published:** 2012-07-03

**Authors:** Abhinav Prakash, Stephen J Parsons, Stuart Kyle, Michael J McPherson

**Affiliations:** 1 Institute of Molecular and Cellular Biology, Faculty of Biological Sciences, University of Leeds, Leeds LS2 9JT, UK; 2 Astbury Centre for Structural Molecular Biology Faculty of Biological Sciences University of Leeds, Leeds LS2 9JT, UK

**Keywords:** Self-assembly, Peptide, Hydrogel, Recombinant expression, Scaffold

## Abstract

**Background:**

Self-assembling peptides that form nanostructured hydrogels are important biomaterials for tissue engineering scaffolds. The P_11_-family of peptides includes, P_11_-4 (QQRFEWEFEQQ) and the complementary peptides P_11_-13 (EQEFEWEFEQE) and P_11_-14 (QQOrnFOrnWOrnFOrnQQ). These form self-supporting hydrogels under physiological conditions (pH 7.4, 140 mM NaCl) either alone (P_11_-4) or when mixed (P_11_-13 and P_11_-14). We report a SUMO-peptide expression strategy suitable for allowing release of native sequence peptide by SUMO protease cleavage.

**Results:**

We have expressed SUMO-peptide fusion proteins from pET vectors by using autoinduction methods. Immobilised metal affinity chromatography was used to purify the fusion protein, followed by SUMO protease cleavage in water to release the peptides, which were recovered by reverse phase HPLC. The peptide samples were analysed by electrospray mass spectrometry and self-assembly was followed by circular dichroism and transmission electron microscopy.

**Conclusions:**

The fusion proteins were produced in high yields and the β-structured peptides were efficiently released by SUMO protease resulting in peptides with no additional amino acid residues and with recoveries of 46% to 99%. The peptides behaved essentially the same as chemically synthesised and previously characterised recombinant peptides in self-assembly and biophysical assays.

## Background

Rationally designed self-assembling peptides have recently attracted widespread attention for the development of novel biomaterials in applications such as tissue engineering scaffolds [1-5] and dental enamel remineralisation [6]. The P_11_-family of peptides comprises over 20 different peptides designed by Aggeli and colleagues to self-assemble into β-sheet structures under various physicochemical conditions to form isotropic hydrogels at peptide concentrations of 10–30 mg/mL [7-9]. These peptides have varying overall charge, hydrophobicity, and polar amino acids resulting in a difference in properties such as solvent affinity, dissolution rate and rigidity of gels.

Chemically synthesised peptide P_11_-4 (CH_3_CO-QQRFEWEFEQQ-NH_2_) is a pH responsive self assembling peptide which forms β-sheets and nematic gels at a concentration of 12.6 mM in water on a pH trigger [7,8] or under physiological conditions in cell culture medium (DMEM) at pH 7.4 [10]. Peptides P_11_-13 (CH_3_CO-EQEFEWEFEQE-NH_2_) and P_11_-14 (CH_3_CO-QQOFOWOFOQQ-NH_2_) are complementary self-assembling peptides that will not self-assemble independently but when combined they assemble with each other to form a hydrogel [11].

Recently there have been reports of recombinant production of self-assembling peptides although there can be issues of low production levels, cell toxicity and degradation by proteases [12-14]. For short peptides <50 amino acids these issues are normally addressed by expressing the peptide as part of a larger ‘fusion partner protein’ to optimise intracellular stability and facilitate affinity isolation during purification. However, there remains the challenge of separating the peptide from its fusion partner.

Previous work on recombinant production of the β-structured P_11_ self-assembling peptides has focused upon a ketosteroid isomerase fusion partner to produce peptides P_11_-4 [10,12] and P_11_-2 [15]. These studies reported a maximal yield of 4.6 g fusion protein/L culture for P_11_-4 [10] by autoinduction [16]. However, the fusion proteins accumulate as insoluble inclusion bodies and peptides are released by denaturation followed by chemical cleavage of a methionine residue with cyanogen bromide [10,12] or a cysteine residue with 1-cyano-4-dimethylaminopyridinium [15]. The use of chemicals for cleavage can lead to problems of disposal on scale-up and results in a non-native peptide sequence. Thioredoxin has also been used as a fusion partner with enzymatic cleavage to release fused peptides, in this case with tobacco etch virus protease [17]. However, this leaves either a Gly or Ser as the N-terminal residue and so usually results in a non-native N-terminus.

In the present study, we have used SUMO (small ubiquitin-related modifier) protein as a fusion partner for various self-assembling P_11_-peptides to address the question, can high yields of self assembling P_11_-family peptide with native termini be produced from a soluble fusion protein system? SUMO fusion technology has been widely used for difficult to express peptides and proteins [18] and due to the nature of cleavage by the SUMO protease, which recognises a structural rather than sequence feature, allows the production of proteins or peptides with any N-terminal residue [19,20]. The approach uses the *S. cerevisiae* SUMO as a fusion partner to allow soluble expression of fusion proteins which can be easily purified using an affinity purification tag. The tertiary structure of SUMO, rather than a sequence motif, is recognised and cleaved by SUMO protease which cleaves after two Gly residues at the C-terminus of SUMO thus releasing the associated protein or peptide with a native N-terminus. SUMO has been successfully used for production of vesicle forming peptides [21] and another self-assembling peptide EAK_16_[22].

We have developed a SUMO-peptide expression strategy suitable for producing soluble fusion proteins and have recovered three different P_11_-family peptides, P_11_-4 and the complementary peptides P_11_-13 and P_11_-14 (K). In the latter case the ornithine in the chemically prepared peptide is replaced by lysine residues. We have characterised these recombinant peptides and show that they behave essentially the same as previously characterised chemically synthesised and recombinant peptides.

## Results

### Cloning and expression strategy

**Figure 1:**
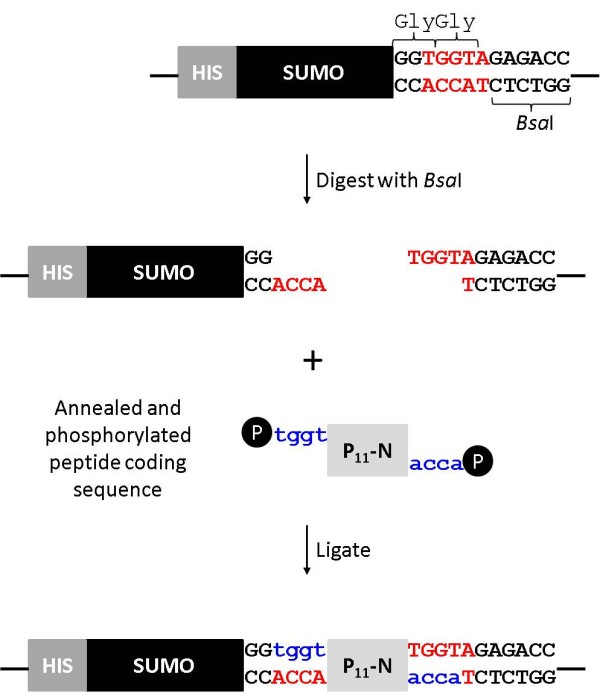
**Cloning strategy outlining the cloning of peptide coding regions at the *Bsa*I restriction site of pET28_ SUMOadapt.**

The pET SUMOadapt vector was kindly provided by Bosse-Doenecke [23]. This modified vector carries an insertion of a multiple cloning site with a *Bsa*I site positioned conveniently to allow the cloning of a coding DNA sequence in-frame with the Gly-Gly motif at the C-terminal cleavage site of SUMO protease [23]. However, the Invitrogen parent vector is based on pBR322 which has a low to moderate copy number. As our objective was to enhance the level of protein production we subcloned the SUMO adapt region into a high copy number pET-28 vector. A PCR reaction was performed using the primers CpRd_NcoI_F and T7 reverse, and the product was cloned into pET28c using restriction enzymes *Nco*I and *Bam*HI. This generated the expression vector pET28_SUMOadapt. Peptide coding sequences, containing a translation termination codon to ensure a native C-terminal end, were cloned into the *Bsa*I site (Figure 1).

A *S. cerevisiae* SUMO protease gene codon-optimised for *E. coli* expression was synthesised by Genscript and was sub-cloned into the expression vector pET11a.

### Expression of SUMO_P_11_-N and SUMO protease by autoinduction

The term P_11_-N is used to represent any of the P_11_ family of peptides. The pET28_SUMOadapt was tested for SUMO protein production to select the optimal growth medium and induction time. Auto-induction trials indicated production of soluble protein using *E. coli * BL21 Star (DE3). Terrific broth (TB) and 8ZY media supplemented with 6% (v/v) 50 X 5052 [16] were tested and TB with 5052 was found to result in a higher cell culture density and level of SUMO production over the growth period tested. The maximum OD_600_ was 45 corresponding to a fusion protein level of 1.5 g/L. A harvest time of 64 hours was selected for maximal soluble protein production.

Optimal yield of soluble SUMO protease was also achieved under these conditions after 64 hours culture.

### Extraction and purification of SUMO_P_11_-N and SUMO Protease

**Figure 2:**
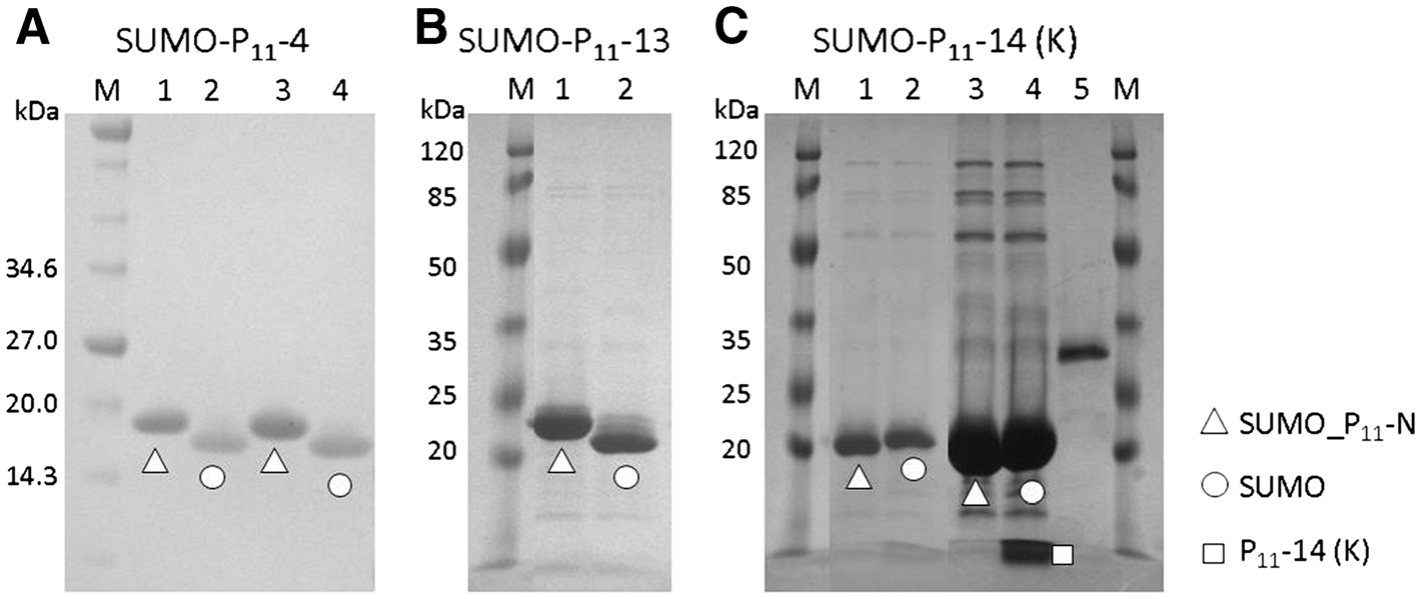
**SDS-PAGE gels showing the cleavage of SUMO_P**_**11**_**-N with SUMO protease. A)** Uncleaved and SUMO protease cleaved SUMO_P_11_-4 in either buffer (lanes 1 and 2) or water (lanes 3 and 4). **B)** SUMO_P_11_-13 uncleaved (lane 1) and SUMO protease cleaved in water (lane 2) and **C)** SUMO_P_11_-14 (K) uncleaved (lane 1) and SUMO protease cleaved in water (lane 2). Lanes 3 and 4 show overloaded samples of Lanes 1 and 2 respectively to allow visualisation the released P_11_-14 (K) peptide (lane 4). Lane 5 shows SUMO protease.

Cells were lysed by cell disruption and centrifuged to recover the soluble fraction. This was filtered and subjected to immobilised metal affinity chromatography (IMAC) purification by batch binding using nickel-nitriloacetic acid (Ni-NTA) resin (Novagen) with batch elution using 250 mM imidazole. A high level of purity was achieved by this single purification step and the fusion proteins were subjected to SUMO protease cleavage. A two hour incubation at 37°C in 1:1000 (SUMO protease:SUMO fusion) mass ratio was sufficient to efficiently cleave the fusion protein releasing the peptide. Figure 2 shows SDS-PAGE results of a SUMO protease cleavage experiment with the three SUMO fusion proteins. The efficiency of cleavage was estimated to be >90% by densitometry. Interestingly cleavage worked as efficiently in water as in cleavage buffer (Figure 2). It is clear that the properties of the peptide influence the SDS-PAGE migration characteristics of the SUMO-peptide fusion proteins. SUMO-P_11_-4 and SUMO-P_11_-13 migrate in a similar manner and upon SUMO protease cleavage the SUMO protein migrates further within the gel. By contrast the positively charged peptide causes the SUMO-P_11_-14(K) to migrate more rapidly than the P_11_-4 or P_11_-13 fusion proteins. However, following SUMO protease cleavage of P_11_-14(K) the SUMO shows an apparent decrease in migration rate to a position corresponding to the cleaved SUMO proteins from the P_11_-4 and P_11_-13 fusion samples.

### Reverse phase HPLC (RP-HPLC)

**Figure 3:**
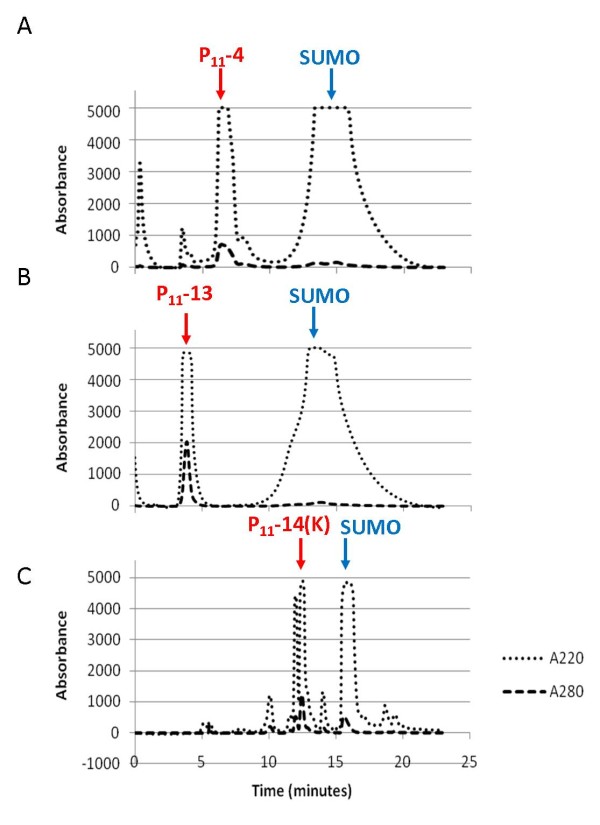
**Average absorbance traces of cleaved SUMO_P**_**11**_**-N when purified using RP-HPLC on a C18 column.** Absorbance measurements were made at 280 nm and 220 nm with the fraction collector programmed to collect peaks at 220 nm. The positions of the peaks subsequently identified to contain the peptide and SUMO protein are indicated by arrows. **A)** P_11_-4 purification with the sharp peak at 6–7 minutes corresponding to P_11_-4 and the broader peak between 11 and 20 minutes corresponding to SUMO. **B)** P_11_-13 purification. **C)** P_11_-14 (K) purification.

Following peptide cleavage it was necessary to separate the peptide from other reaction components by RP-HPLC. The cleavage reaction mixture was adjusted to pH 9.0 with NH_4_OH and incubated overnight before filtering (0.22 μm) prior to injection onto a C18 column. The elution profile was monitored at 220 and 280 nm and Figure 3 shows chromatograms of typical separations. The fractions were collected using the 220 nm absorbance setting rather than 214 nm due to a limitation with the fraction collector. The fractions corresponding to distinct peaks were collected separately and analysed by mass spectrometry. The peptides were in the major peaks eluting between 4 and 8 minutes and were pooled and lyophilised. The difference in retention time for P_11_-14 and associated SUMO compared with the P_11_-4 and P_11_-13 samples is most likely due to the alternative buffer system used for this positively charged peptide.

### Mass spectrometry characterisation

To confirm the identity of the fusion proteins and peptides electrospray mass spectrometry was performed. Fusion proteins were dialysed overnight against 50 mM ammonium bicarbonate (pH 8.0) using Spectra/POR 6 dialysis 1 kDa cut off membrane (Spectrum laboratories). Peptide samples were lyophilised. The theoretical mass of the fusion proteins were calculated by Expasy Protoparam for protein samples lacking the N-terminal methionine and the masses measured by mass spectrometry are shown in Table 1. The masses were in excellent agreement confirming the identities of the fusion proteins and the peptides.

**Table 1:** Comparison of calculated and mass spectrometry determined molecular masses of fusion protein and peptide samples

**Protein/peptide sample**	**Calculated mass (Da)‡**	**Observed mass (Da)**	**Difference (Da)**
SUMO_P_11_-4	14821.4	14820.8	−0.6
SUMO_P_11_-13	14796.3	14796.4	+0.1
SUMO_P_11_-14 (K)	14790.5	14789.6	−0.9
SUMO cleaved	13284.8	13284.0	−0.8
P_11_-4	1554.6	1553.7	−0.9
P_11_-13	1529.5	1528.6	−0.9
P_11_-14 (K)	1523.8	1522.8	−1.0

‡ The calculated molecular mass is of SUMO protein lacking the N-terminal methionine residue.

 Electrospray MS-MS sequencing confirmed the identity of nine of the eleven amino acids in each of the peptides.

### Peptide quantification

UV spectroscopy was used to quantify the peptides based on their theoretical extinction coefficients. SUMO protein has 118 amino acids excluding the N-terminal methionine, while the peptides comprise 11 amino acids, representing between 10.3 and 10.7% of the mass of the fusion proteins. In an experiment to compare the yields of each peptide, a series of parallel purifications were performed and the results are shown in Table 2. These show that the peptides were well purified, with yields of 99.6% for P_11_-13, 84% for P_11_-4 and 46.1% for P achieved. The reason for the lower yield for P_11_-14 may be due to the different buffer system or the positively charged nature of the peptide. The yield of 46% is good but further work is required to determine the underlying reason for the lower recovery and to try to optimise recovery of this peptide.

**Table 2:** Yields of purified SUMO fusion proteins and peptides

**Peptide**	**Fusion protein yield (mg/L)**	**Peptide as percentage mass of fusion protein (%)**	**Theoretical yield of peptide (mg/L)**	**Actual yield of peptide purified (mg/L)**	**Percent theoretical yield (%)**
P_11_-4	400	10.49	42.0	35.3	84.0
P_11_-13	500	10.34	51.7	51.5	99.6
P_11_-14	375	10.30	38.6	17.8	46.1

### Formation of peptide hydrogels

**Figure 4:**
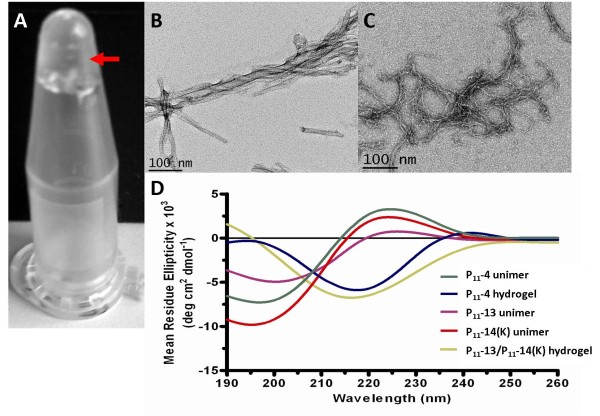
**Hydrogel analysis of P**_**11**_**-13/P**_**11**_**-14. A) **Hydrogel formed upon equimolar mixing of P_11_-13 and P_11_-14(K) indicated by arrow. B and C) Transmission electron microscopy (TEM) images of self-assembled **B)** P_11_-4 at pH 2 and **C)** P_11_-13/P_11_-14(K)**. D)** Circular dichroism analysis of P_11_-4, P_11_-13 and P_11_-14(K) unimers, and P_11_-4 and P_11_-13/P_11_-14(K) hydrogels at pH 7.4. Random coil conformation is observed for peptide unimers while a β-sheet conformation is observed for peptide hydrogels.

The purified peptides were dissolved in 140 mM NaCl to a final concentration of 10 mg/mL. The pH of the P_11_-4 solution was adjusted to ca. 2.0 to trigger formation of a self supporting gel. P_11_-13 and P_11_-14 were also prepared to a final concentration of 10 mg/mL at ca. pH 7 and were mixed in equal volumes to form a self supporting gel. Figure 4A shows the hydrogel state for P_11_-13/P_11_-14 when combined.

### Characterisation of peptide fibril formation

Aggeli and colleagues reported the morphological structure of chemically synthesised P_11_-4 fibrils by transmission electron microscopy (TEM) [7,8]. Self supporting gels for P_11_-4 and P_11_-13/P_11_-14 were diluted to 100 μM in distilled water at pH 2.0 or 7.4 respectively. Fibril morphologies were observed by TEM using uranyl acetate negative staining. Figure 4B and C show the intertwining of fibrils creating fibres for P_11_-4 and P_11_-13/P_11_-14(K) hydrogels, respectively. Both long and short fibre structures were observed as isolated structures as well as entangled masses. These are similar to structures that have been previously observed with other samples of these peptides [7,8,10,12].

The secondary structure of the peptide samples was examined as a function of pH using circular dichroism. Peptide hydrogels were diluted to 100 μM solutions. Monomeric forms were also prepared as controls. The results as shown in Figure 4D confirm the random coil conformation of the monomeric forms of P_11_-4, P_11_-13 and P_11_-14(K), and the β-sheet conformations for the P_11_-4 and P_11_-13/P_11_-14(K) hydrogels.

## Discussion

Our previous work led to the development of *E. coli* expression systems capable of producing large amounts of short self-assembling peptides of up to 370 mg/L culture [10,12]. These were produced in the form of inclusion bodies with recovery of the peptides from their fusion partner by urea solubilisation and chemical cleavage. We were interested to explore the extent to which we could express the P_11_-family of β-structured peptides in a soluble format through the use of soluble peptide fusions. We chose to test the SUMO system as it enhances the solubility of proteins and has been effective for recombinant production of difficult proteins with good yields and efficient purification [19,24-27]. Moreover, SUMO protease is highly efficient and does not alter the target protein sequence thereby producing native protein of high quality [28]. To enhance the level of protein and peptide production we required high levels of SUMO expression and therefore subcloned the SUMO adapt region from pET SUMO adapt [23] into a pET28c vector. Constructs were transformed into *E. coli* strain BL21 (DE3) Star and TB-5052 media was used for expression of protein. SUMO-peptide fusions were recovered at 0.37 to 0.5 g/L with HPLC purification from the SUMO protease cleavage reaction resulting in yields of soluble P_11_-peptides of between 18 and 35 mg/L demonstrating that we have achieved good levels of recovery of purified peptides. It is likely this can be increased significantly as the maximum yield of SUMO protein achieved was 1.5 g/L compared to 637 mg/mL reported by Li et al., [28] using a similar expression strategy, while for EAK16 peptide production 250 mg/L of fusion protein was reported by Satakarni et al., [22] by IPTG induction.

Cleavage of fusion protein by SUMO protease was highly efficient in both cleavage buffer and in water. The possibility of performing cleavage reactions in water further demonstrates the efficiency of the system allowing largely salt free solutions containing peptides to be obtained thus reducing difficulties in downstream removal of salts prior to subsequent steps. It is interesting that the SUMO protease works so efficiently in the presence of a low concentration of reducing agent required for cysteine protease function. The dithiothreitol (DTT) concentration in the protease stock solution is 0.5 mM so upon dilution by up to 10,000-fold in water for the cleavage reaction the final concentration would be 50 nM.

We have previously characterised both chemically and recombinantly produced P_11_- peptides [7,8,10-12]. In this study TEM showed that rP_11_-4 formed fibrils with lengths and widths similar to those reported for cP_11_-4 [7,8,10,12] and to our previously purified rP_11_-4 and rP_11_-4(hsl) [10,12] and cP_11_-13/cP_11_-14 similar to those previously observed [11]. Circular dichroism analysis suggested that the predominant secondary structure of rP_11_-4 and P_11_-13/P_11_-14 hydrogels at physiological pH (pH 7.4) was antiparallel β-sheet as has been observed previously [7,8,10-12]. Upon adjusting rP_11_-4 to pH 9, a conformational transition to random coil occurred while solutions of rP_11_-13 and rP_11_-14 alone also displayed random coil conformation. We have previously demonstrated that these P_11_- peptides are cytocompatible with human primary fibroblasts [10,11].

## Conclusions

We have demonstrated the use of a SUMO fusion approach for efficient production and purification of β-structured recombinant self assembling peptides with native N- and C-termini. We have demonstrated that SUMO protease displays efficient cleavage of the fusion protein in water with a very low concentration of reducing agent (50 nM) . The efficiency of SUMO protease cleavage in water can be exploited to produce essentially salt and ion-free solutions of various proteins/peptides for subsequent processing. The purified recombinant peptides behave similarly to chemically synthesized versions. Whilst recombinant production has the potential to produce large quantities of self-assembling peptides for tissue engineering and industrial applications, the greatest benefits are likely to derive from the soluble expression of such peptides as fusions to a range of functional peptide and protein domains to impart tailored biological functions within self-assembled peptidic biomaterials.

## Methods

### Bacterial strains, plasmids and cell culture media

*E. coli strain* XL1 Blue (Stratagene, La Jolla, CA.) was used for routine cloning while BL21 Star (DE3) Star (Invitrogen, Carlsbad, CA) was used for expression. The expression vectors, pET 28c+ and pET 11a+ (Novagen) were used to express the SUMO fusion construct and SUMO protease respectively. pET SUMOadapt was a gift from Dr E. Bosse-Doenecke [23]. A codon optimised SUMO protease gene was synthesised by Genscript and delivered in a pUC 57 plasmid.

The growth media Luria Burtani broth (LB), 8ZY and LB agar were prepared according to [29]. Terrific Broth (TB) was purchased from HiMedia. Auto-induction media were prepared according to the methods of Studier [16] and were supplemented by 6% (v/v) 50 X 5052 supplements. 5052 supplement corresponds to 0.5% (v/v) glycerol, 0.05% (w/v) glucose and 2% (w/v) lactose. Antibiotics carbenicillin and kanamycin were added to media to final concentrations of 100 μg/mL and 50 μg/mL respectively.

### Generation of recombinant E. coli strains

#### Generation of pET28_SUMO fusion construct

The SUMO protein coding region was PCR amplified from pET SUMOadapt [23] with an NcoI site at the 5’ end and a BamHI site at the 3’ end to allow cloning into pET28c . A 50 μL PCR reaction containing 0.5 ng of template DNA (pET SUMOadapt), 15 pmoles each of CpRd_NcoI_F ( ) and T7 terminator primer ( ), 0.2 mM dNTPs, 1mM MgSO4, 1 X KOD buffer, 1 U KOD Hot Start Polymerase (Novagen) was subjected to 1 minute at 95°C, 36 cycles of 30 seconds at 95°C, 30 seconds at 55°C and 1 minute extension at 72°C followed by a final extension for 10 minutes at 72°C. PCR products were analysed on a 1.5% agarose gel and the correct product was purified using a QIAquick PCR clean up kit (Qiagen cat. # 28104). This DNA fragment was digested with NcoI and BamHI and purified as before. A 15 μL reaction containing 1.2 μg of the pET28c vector was digested with 20 units of NcoI and BamHI followed by dephosphorylation with 10 units of Antarctic phosphatase (NEB cat. #M0289L) and the vector backbone was gel extracted (QIAquick Gel Extraction kit cat. #28704). A 10 μL ligation reaction was set up containing 50 ng of pET28c vector backbone, 100 ng of SUMO insert, 10 units of T4 DNA ligase and 1 X T4 DNA ligase buffer.

### Generation of rP_11_-N coding regions

Complementary oligonucleotides encoding self-assembling peptides P_11_-4 (QQRFEWEFEQQ), P_11_-13 (EQEFEWEFEQE) and P_11_-14(K) (QQKFKWKFKQQ) were designed to have a stop codon (bold) and 5’ TGGT or ACCA single strand overhangs (underlined);

P_11_-4 F 5’-tggtcagcagcgctttgaatgggaatttgaacagcag**taa**-3’

P_11_-4 R 5’-acca**tta**ctgctgttcaaattcccattcaaagcgctgctg-3’

P_11_-13 F 5’-tggtgaacaggaatttgaatgggaatttgaacaggaa**taa**-3’

P_11_-13 R 5’-acca**tta**ttcctgttcaaattcccattcaaattcctgttc-3’

P_11_-14 (K) F 5’-tggtcagcagaaatttaaatggaaatttaaacagcag**taa**-3’

P_11_-14 (K) R 5’-acca**tta**ctgctgtttaaatttccatttaaatttctgctg-3’

The SUMOadapt region was designed by Bosse-Doenecke [23] to exploit the *Bsa*I restriction site for cloning. In this case the ends created are ACCA allowing peptide insertion immediately adjacent to the C-terminal Gly-Gly motif (Figure 1).

Oligonucleotides were dissolved to a concentration of 100 pmol/μl in distilled water. A 15 μL phosphorylation reaction was set up at 37°C for 30 min containing 600 pmol of oligonucleotide, 5 U of T4 polynucleotide kinase (NEB cat. #M0236L) and 1 X T4 DNA ligase buffer. The reaction mixture was heat inactivated at 65°C for 20 minutes before 400 pmol of phosphorylated forward and reverse oligonucleotides were mixed with 20 μl of 10 X annealing buffer (400mM Tris HCl pH 8.0, 100mM MgCl_2_ and 500 mM NaCl) in a final volume of 200 μl. The mixture was heated at 99°C for 10 minutes and allowed to cool down slowly to 50°C to allow the complementary sequences to anneal.

### Generation of fusion proteins

pET28_SUMOadapt was digested with *Bsa*I and dephosphorylated using Antarctic phosphatase. Following a QIAquick clean-up, a ligation reaction containing 50 ng of this DNA, 100 ng of peptide encoding DNA fragment, 1 μl of 10X ligase buffer (NEB) and 1 μl of T4 DNA ligase (NEB) was incubated at room temperature for 4 hours. Following transformation of XL1-Blue cells, colonies were screened by colony PCR using Go-Taq Polymerase (Promega) and T7 forward and reverse primers to identify recombinants which were then sequenced to confirm their integrity.

### Generation of SUMO protease expression construct

A His-SUMO protease [30] codon optimised DNA sequence was synthesised by Genscript in a pUC57 vector. This was subcloned into pET 11a digested with *Nde*I and *Bam *HI and dephosphorylated. Colony PCR was used to screen transformants and the coding region was verified by DNA sequencing.

### Fusion protein expression

Protein expression studies were performed using the auto-induction protocol developed by Studier [16]. A single colony was used to inoculate 2 mL of TB media containing antibiotic with growth at 37°C for 6 hours at 250 rpm. A 250 μl aliquot of the culture was used to inoculate 400 mL of TB-5052 auto-induction media in a 2L flask and incubated at 25°C at 250 rpm. For time course experiments, 1 mL samples were collected at time intervals up to 64 h. Cells were harvested at 64 h post inoculation by centrifugation at 6000 rpm for 20 min using fixed angle rotor (Sorvall SLA1500).

### Preparation of soluble proteins from *E. coli* cultures

Cell pellets were re-suspended in extraction buffer (50 mM NaH_2_PO_4_, 300 mM NaCl, 20 mM imidazole, pH 8.0) supplemented with protease inhibitor cocktail (Complete EDTA free protease inhibitors) (1 tablet/40 mL) and 20 Units of Omnicleave endonuclease. The cell suspension was then subjected to two cycles of mechanical disruption at 30,000 psi using a cell disruptor (Constant Cell Disruption Systems model 2 PLUS). Following cell lysis, the soluble fraction was isolated by centrifugation at 13,000 x *g* for 45 minutes to pellet the insoluble fraction. The supernatant (soluble phase) was removed, filtered through a 0.22 μm filter (Sartorius) and stored at −80°C.

### SUMO_peptide purification by immobilised IMAC

The Novagen ‘Batch Purification of 6X His-tagged proteins from *E. coli* under native conditions’ protocol was followed. The soluble fractions were filtered with a 0.45μm filter (Sartorius) and loaded onto a pre-equilibrated Ni-NTA column. Columns were then washed with extraction buffer containing 50 mM then 100 mM imidazole and proteins were eluted in elution buffer (50 mM NaH_2_PO_4_, 300 mM NaCl, 250 mM imidazole, pH 8.0). Proteins were concentrated using centrifugal filters (Amicon Ultra, Millipore) and buffer exchanged in to either PBS or dH_2_O prior to SUMO protease cleavage. This exchange was achieved by repeated dilution and centrifugation with the required buffer to remove trace salts.

### SDS-PAGE analysis

Protein samples were mixed in SDS loading buffer (12% SDS, 6% mercaptoethanol, 30% Glycerol, 0.05% bromophenol blue in 1M Tris–HCl (pH 6.8) in a 4:1 (v/v) ratio of sample to loading buffer and heated at 95°C for 5 minutes. Samples were analysed using 4–12% NuPAGE Novex Bis-tris precast gels (Invitrogen, UK) or home-made 12% SDS-PAGE gels at 150 V for 1 hour. Protein bands were visualized using Coomassie Blue G-250 (BDH Chemicals) or Simply Blue^TM^ Safe stain (Invitrogen, UK).

### Protein concentration determination

Protein concentrations were determined either by Bradford assay (Bio-Rad cat. 500–0201) or UV spectroscopy. Theoretical extinction coefficients were calculated using ProtParam (http://ca.expasy.org/cgi-bin/protparam) to estimate the concentration according to Beer-Lambert Law. The extinction coefficients for SUMO was 1490 M^-1^ cm^-1^, P_11_-4 was 5500 M^-1^ cm^-1^ while SUMO protease was 30035 M^-1^ cm^-1^.

### SUMO protease expression, purification and storage

SUMO protease was expressed by autoinduction in *E. coli* BL21 Star (DE3) and harvested after 64 hours. Cell pellets were resuspended in binding buffer (50 mM NaPi, 300 mM NaCl, 20 mM imidazole, pH 8.0) supplemented with Omnicleave endonuclease. SUMO protease was purified by IMAC and immediately following elution, DTT was added to a concentration of 1 mM and the purified SUMO protease was dialysed against phosphate buffered saline (PBS; 50 mM NaPi, 300 mM NaCl, pH 8.0) with 1 mM DTT. This was concentrated and quantified using a Bradford assay as 5 mg/mL. Finally glycerol was added to 50% (v/v) and the SUMO protease was stored at −80°C until required.

### Cleavage of SUMO_peptide fusion protein with recombinant SUMO protease

SUMO protease was used in a 1:1000 molar ratio for 2 hours or 1:10,000 molar ratio overnight for cleavage of SUMO fusion protein. The reaction was carried out in phosphate buffered saline (PBS) buffer (pH 8.0) in presence of 1mM DTT or in water without DTT and incubated at 37°C. SDS-PAGE analysis was performed to verify the cleavage.

### Reversed phase high performance liquid chromatography (RP HPLC)

A Dionex Summit machine using Chromeleon software for peak analysis was used for reverse phase high performance liquid chromatography (RP HPLC). A C 18 (Octadecyl derivatized silica) semiprep column was used at room temperature to separate various peaks. P_11_-4 and P_11_-13 were purified using Buffer A (5% acetonitrile/95% water, pH 9.0) and Buffer B (95% acetonitrile/5% water, pH 9.0) which were adjusted to pH 9.0 using ammonium hydroxide (NH_4_OH) to maintain the peptides’ unimeric conformation. Peptide P_11_-14 (K) was purified with NH_4_OH, 0.1% and 0.06% trifluoroacetic acid (TFA) added to Buffer A and Buffer B respectively. All buffers were filtered through a 0.2 μm filter prior to use. Samples were filtered using 0.45 μm filter prior to loading. The semiprep column was initially equilibrated with Buffer A at a flow rate of 2.0 mL/min and a blank run was performed. Following sample injection at t = 0, a gradient elution was performed from 0 – 90% Buffer B over 20 minutes. The elution fractions were analysed using absorbance measurement at 280 nm and 220 nm. The fraction collector was programmed to collect peaks from 220 nm absorbance. The process was repeated for multiple injections to purify maximum amount of peptide and peptide containing peaks were collected, pooled and lyophilized before being submitted for mass spectrometry as a powder.

### Mass spectrometry

Protein samples were dialysed against excess 50 mM ammonium bicarbonate (pH 8.0) or dH_2_O overnight and peptide samples were prepared by dissolving HPLC purified lyophilised powder in 20 μL of methanol containing 1 μL of 100% formic acid. Samples were submitted to the Mass Spectrometry Facility, Astbury Centre, University of Leeds and analysed by Dr. James Ault on a Synapt HDMS (Waters UK Ltd.) mass spectrometer. Peptide samples were subsequently sequenced by tandem mass spectrometry (MS-MS).

### Formation of peptide hydrogels

Lyophilised peptide samples were dissolved in 140 mM NaCl solution at pH 7.4 to a concentration of 10 mg/mL. To unimerise the peptides, the pH was adjusted using 1 M NaOH and 1 M HCl (high pH for negative P_11_-4 and P_11_-13 and low pH for P_11_-14 (K)). Self-assembly was induced in P_11_-4 samples by reducing the pH below pH 7.0. For P_11_-13 and P_11_-14 (K) complementary-assembly was achieved by mixing equimolar volumes.

### Transmission electron microscopy

Self-assembled peptide gels of P_11_-4 and of P_11_-13/P_11_-14 (K) were formed at 6.3 mM as described above. Following overnight incubation at room temperature, these were diluted with double distilled water to 100 μM. The morphology of the resulting fibrils was visualized by TEM using uranyl acetate negative staining. Glow-discharged, carbon coated 400 hexagonal mesh copper grids were activated by UV light for 20 minutes. Grids were covered with 20 μl of peptide solution and allowed to adsorb for 1 minute. Excess sample was drained with filter paper and grids were negatively stained using 10 μl of 4% uranyl acetate solution (w/v in water) for 20 seconds. Excess solution was removed using filter paper and grids were allowed to air dry before TEM analysis. Images were obtained using a Jeol 1200 EX TEM operating at 80 kV. Peptide hydrogels were analysed in duplicate.

### Circular dichroism of recombinant peptides

Peptide gels were diluted to 100 μM in water as described above. Samples were loaded in quartz cuvettes (Hellma) with a path length of 1 mm. Mean residual ellipticity readings were taken at far UV region of spectrum (190 nm to 260 nm) using a Jasco J-750 spectropolarimeter. Each spectrum was the average of 8 scans with a step resolution of 0.5 nm, scan speed 50 nm.min^-1^, response time of 1 second and a sensitivity of 50 m° at 20°C. Blank readings were taken for all samples and subtracted from the peptide samples results

## Abbreviations

DTT: Dithiothreitol; IMAC: Immobilised metal affinity chromatography; Ni-NTA: Nickel-nitriloacetic acid; Orn: Ornithine; RP-HPLC: Reverse phase HPLC; SDS-PAGE: Sodium dodecyl sulphate-polyacrylamide gel electrophoresis; SUMO: Small ubiquitin-related modifier; TB: Terrific broth; TEM: Transmission electron microscopy.

## Competing interest

The authors declare that there is no competing interest involved.

## Authors’ contribution

AP and SJP carried out the experiments. SK advised and analysed CD and TEM experiments. MJM conceived, designed and co-ordinated the study and was involved in data analysis. All authors were involved in the production and review of the manuscript and have read and approved the final manuscript.
